# OPC-67683, a Nitro-Dihydro-Imidazooxazole Derivative with Promising Action against Tuberculosis In Vitro and In Mice

**DOI:** 10.1371/journal.pmed.0030466

**Published:** 2006-11-28

**Authors:** Makoto Matsumoto, Hiroyuki Hashizume, Tatsuo Tomishige, Masanori Kawasaki, Hidetsugu Tsubouchi, Hirofumi Sasaki, Yoshihiko Shimokawa, Makoto Komatsu

**Affiliations:** 1 Microbiological Research Institute, Otsuka Pharmaceutical, Tokushima, Japan; 2 Medicinal Chemistry Research Institute, Otsuka Pharmaceutical, Tokushima, Japan; 3 Tokushima Research Institute, Otsuka Pharmaceutical, Tokushima, Japan; University of California San Francisco, United States of America

## Abstract

**Background:**

Tuberculosis (TB) is still a leading cause of death worldwide. Almost a third of the world's population is infected with TB bacilli, and each year approximately 8 million people develop active TB and 2 million die as a result. Today's TB treatment, which dates back to the 1970s, is long and burdensome, requiring at least 6 mo of multidrug chemotherapy. The situation is further compounded by the emergence of multidrug-resistant TB (MDR-TB) and by the infection's lethal synergy with HIV/AIDS. Global health and philanthropic organizations are now pleading for new drug interventions that can address these unmet needs in TB treatment.

**Methods and Findings:**

Here we report OPC-67683, a nitro-dihydro-imidazooxazole derivative that was screened to help combat the unmet needs in TB treatment. The compound is a mycolic acid biosynthesis inhibitor found to be free of mutagenicity and to possess highly potent activity against TB, including MDR-TB, as shown by its exceptionally low minimum inhibitory concentration (MIC) range of 0.006–0.024 μg/ml in vitro and highly effective therapeutic activity at low doses in vivo. Additionally, the results of the post-antibiotic effect of OPC-67683 on intracellular Mycobacterium tuberculosis showed the agent to be highly and dose-dependently active also against intracellular M. tuberculosis H37Rv after a 4-h pulsed exposure, and this activity at a concentration of 0.1 μg/ml was similar to that of the first-line drug rifampicin (RFP) at a concentration of 3 μg/ml. The combination of OPC-67683 with RFP and pyrazinamide (PZA) exhibited a remarkably quicker eradication (by at least 2 mo) of viable TB bacilli in the lung in comparison with the standard regimen consisting of RFP, isoniazid (INH), ethambutol (EB), and PZA. Furthermore, OPC-67683 was not affected by nor did it affect the activity of liver microsome enzymes, suggesting the possibility for OPC-67683 to be used in combination with drugs, including anti-retrovirals, that induce or are metabolized by cytochrome P450 enzymes.

**Conclusions:**

We concluded that based on these properties OPC-67683 has the potential to be used as a TB drug to help combat the unmet needs in TB treatment.

## Abstract 2

A nitro-dihydro-imidazooxazole derivative was shown to have the potential for use against tuberculosis.

## Editors' Summary

**Background.:**

One-third of the world's population is infected with *Mycobacterium tuberculosis,* the bacterium that causes tuberculosis (TB). Most infected people are healthy—the bacteria can remain latent for years, hidden within cells in the body. However, every year 8 million people develop active TB, a chronic disease that usually affects the lungs, and 2 million people die. For most of the second half of the 20th century, TB was in decline because of the powerful antibiotics that were developed from the 1940s onwards. The standard treatment for TB—four antibiotics that have to be taken several times a week for at least six months to flush out any latent M. tuberculosis bacteria—was introduced in the late 1970s and saved many lives. Recently, however, efforts to eradicate TB have been set back by the HIV/AIDS epidemic—people with damaged immune systems are very susceptible to TB—and the emergence of multi-drug resistant (MDR) bacteria.

**Why Was This Study Done?:**

The treatment for TB is long and unpleasant, and patients who develop MDR-TB have to be treated with second-line drugs that are less effective, more expensive, and more toxic. In addition, for people infected with both HIV and TB, some antiretroviral and anti-TB drugs cannot be used at the same time. Many drugs are either activated or removed by enzymes in the liver, so combinations of these two classes of drugs sometimes alter liver function in a way that causes clinical problems. There is, therefore, an urgent need for new, effective anti-TB drugs that attack M. tuberculosis in a different way than do existing drugs. Such drugs should ideally be active against MDR M. tuberculosis, work quickly at low doses, be active against latent bacteria, and have minimal effects on the liver so that they can be used in patients co-infected with HIV. In this study, the researchers investigated a chemical called OPC-67683.

**What Did the Researchers Do and Find?:**

The researchers identified a compound that inhibited the production of mycolic acid—an essential component of the cell wall of *M. tuberculosis*—and they tested its ability to kill the organism. They then tested in detail its ability to inhibit bacterial growth in dishes of antibiotic-sensitive and MDR *M. tuberculosis* and isolates from patients. OPC-67683 inhibited the growth of all these bugs at lower concentrations than the four antibiotics used in the standard TB treatment. It also killed bacteria hidden within human cells as well as or better than these drugs. Next, the researchers treated mice infected with M. tuberculosis with OPC-67683. They found that it reduced the number of bacteria in the lungs of both normal and immunocompromised mice at lower concentrations than the standard drugs. Furthermore, when combined with two of the standard drugs, it reduced the time taken to clear bacteria from the lungs by the standard drug regimen by two months. Finally, the researchers showed that OPC-67683 had no effects on the liver enzymes that metabolize antiretrovirals, and, conversely, that the activity of OPC-67683 was not affected by liver enzymes. Thus, this agent is unlikely to cause clinical problems or lose its efficacy in HIV patients who are receiving antiretroviral drugs.

**What Do These Findings Mean?:**

These results from laboratory and animal experiments suggest that OPC-67683 could possibly fulfill the criteria for a new anti-TB drug. OPC-67683 is active against MDR-TB. It is also active against intracellular TB, which the authors postulate could be a positive link with the effective treatment of latent TB, and it works quickly in animals when combined with existing anti-TB drugs. Importantly, it also disables M. tuberculosis in a unique way and does not appear to have any major effects on the liver that might stop it from being used in combination with antiretrovirals. All these preclinical characteristics now need to be checked in people—many drugs do well in preclinical studies but fail in patients. These clinical studies need to be expedited given the upsurge in TB, and, write the researchers, OPC-67683 needs to be tested in combination with both conventional drugs and other new drugs so that the best regimen of new drugs for the treatment of TB can be found as soon as possible.

**Additional Information.:**

Please access these Web sites via the online version of this summary at http://dx.doi.org/10.1371/journal.pmed.0030466.

- US National Institute of Allergy and Infectious Diseases patient fact sheet on tuberculosis
- US Centers for Disease Control and Prevention information on tuberculosis
- MedlinePlus encyclopedia entry on tuberculosis
- NHS Direct Online patient information on tuberculosis from the UK National Health Service
World Health Organization information on the global elimination of tuberculosis
- Global Alliance for TB Drug Development information on why new TB drugs are needed

## Introduction

Tuberculosis (TB) is still a leading cause of death worldwide [1]. Almost a third of the world's population is infected with TB bacilli, and each year approximately 8 million people develop active TB and 2 million die as a result [2]. Today's TB treatment, which dates back to the 1970s, is long and burdensome, requiring at least 6 mo of multidrug chemotherapy, typically consisting of rifampicin (RFP), isoniazid (INH), ethambutol (EB), and pyrazinamide (PZA) given under clinically observed conditions. The situation is further complicated by the emergence of multidrug-resistant TB (MDR-TB) and by the infection's lethal synergy with HIV/AIDS [3–6]. Patients with MDR-TB must be treated with a combination containing second-line drugs that are less effective, more expensive, and more toxic. TB's lethal synergy with HIV/AIDS puts HIV-positive individuals with latent tubercle bacilli infection (LTBI) at a 30× to 50× greater risk of developing active TB, giving rise to TB as the number one killer among patients with AIDS [6].

The pharmaceutical industry, however, has generally shown little interest in developing new, more effective drugs to address these needs, and, as a result, no new anti-TB agent with a novel mechanism of action has been launched since the introduction of RFP in 1966. Consequently, global health and philanthropic organizations are now pleading for new chemotherapy interventions that can shorten the total duration of therapy, provide improved efficacy against MDR-TB, safely treat patients co-infected with HIV/AIDS, and target LTBI [6,7].

**Figure 1:**
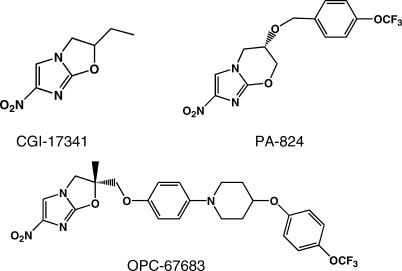
Structure of CGI-17341, PA-824, and OPC-67683 OPC-67683: (*R*)-2-methyl-6-nitro-2-(4-[4-(4-trifluoromethoxyphenoxy)piperidin-1-yl]phenoxymethyl}-2,3-dihydroimidazo[2,1-*b*]oxazole.

We initiated a program to screen for potent anti-TB agents that have a new structure and mechanism able to inhibit the biosynthesis of mycolic acid, and found nitro-dihydro-imidazooxazole derivatives to exhibit such activity. Nitro-heterocyclic compounds, including various 5- and 2-nitroimidazoles and 5-nitrofurans, are known to be effective against a variety of protozoan and bacterial infections in humans and animals [8]. These compounds, however, are also known to commonly possess mutagenicity. CGI-17341 (Figure 1), a nitroimidazooxazole derivative, has been reported to have anti-tubercular activity [9,10], but the compound was not developed because of its mutagenic properties. We focused our search on new nitro-dihydro-imidazooxazoles with anti-tubercular activity that had no mutagenicity by performing the bacterial reverse mutation (BRM) test [11]. About 95% of the compounds we screened earlier that had mono- or di-alkyl substituents at 2-position were mutagenic. However, after introducing heteroatoms to the substituent, we were able to successfully decrease the mutagenicity rate to 16%. Among the non-mutagenic derivatives, we found OPC-67683 to have potent anti-TB activity. We then further evaluated OPC-67683 to determine whether the compound could help address the unmet needs of TB treatment.

## Methods

### Culture Medium

Cultures of Mycobacterium tuberculosis and M. bovis BCG were grown in Middlebrook 7H9 broth (BBL, http://www.bd.com) and Middlebrook 7H11 agar medium (BBL), respectively. Both types of media were prepared according to the manufacturer's directions.

### Drug Preparation for In Vitro Studies

OPC-67683, PA-824, and CGI-17341 were synthesized at Otsuka Pharmaceutical (http://www.otsuka.global.com); RFP, INH, EB, streptomycin (SM), and PZA were purchased from Sigma (http://www.sigmaaldrich.com). OPC-67683, RFP, INH, PZA, and PA-824 were each dissolved in dimethylsulfoxide (DMSO), and the solutions were diluted serially with DMSO in 2-fold dilutions to desired concentrations. EB and SM were dissolved in distilled water, and the solutions were serially diluted with distilled water in 2-fold dilutions to desired concentrations.

### Drug Preparation for In Vivo Studies

OPC-67683, PA-824, RFP, INH, EB, and PZA were each pestled in a mortar and dissolved or suspended in 5% gum arabic solution using an ultrasonic generator. Two-fold dilutions were then conducted using 5% gum arabic solution to adjust to the desired concentrations.

### Strains

M. tuberculosis ATCC 25618 (H37Rv), *M. tuberculosis* ATCC 35838 (H37Rv-R-R), M. tuberculosis ATCC 35822 (H37Rv-H-R), M. tuberculosis ATCC 35837 (H37Rv-E-R), M. tuberculosis ATCC 35820 (H37Rv-S-R), M. tuberculosis ATCC 35801 (Erdman), and *M. tuberculosis* ATCC 35812 (Kurono) were purchased from American Type Culture Collection (http://www.atcc.org). M. bovis IID 982 (BCG Tokyo) was purchased from the Institute of Medical Science, University of Tokyo. A total of 67 M. tuberculosis strains used in this study were isolated in Japan, Myanmar, Thailand, Cambodia, Indonesia, Vietnam, and China.

### BRM Test

The BRM test was performed in accordance with OECD Guideline 471 using Salmonella tyiphimurium TA98, TA100, TA1535, and TA1537, and Escherichia coli WP2 *uvrA* [11]. Each bacterial strain was pre-cultured at 37 °C for 18 h using a nutrient broth (Nissui Pharmaceutical; http://www.nissui-pharm.co.jp/index_e.html). After adjustment to 2.4 at OD660 nm, each bacterial suspension was added to a test tube containing the designated compound in the absence or presence of rat liver microsome (S9) mix. After a 20-min incubation at 37 °C, top agar was added to each test tube and the contents were poured into minimum essential medium (Oriental Yeast; http://www.oyc.co.jp/e/index.htm). The number of revertants was counted 48 h after incubation at 37 °C.

### Susceptibility Testing

Susceptibility testing was performed using a procedure previously reported [12,13]. Bacteria stocks preserved in a deep freezer were each dissolved and adjusted to approximately 10^6^ colony-forming units (CFU)/ml. Drug-containing plates were inoculated with the designated bacterial suspension to approximately 10^6^ CFU/ml using a multipoint inoculator (Sakuma Seisakusho; http://homepage1.nifty.com/sakuma2000). Each plate was incubated at 37 °C for 14 d and analyzed to determine the minimum inhibitory concentration (MIC). The MIC was expressed as the lowest concentration that inhibited visible growth of organism on the agar medium after incubation.

For the evaluation of susceptibility against clinically isolated strains, resistance was determined based on the following criteria recommended by the National Committee for Clinical Laboratory Standards [14]: 1.0 μg/ml for RFP, 1.0 μg/ml for INH, 7.5 μg/ml for EB, and 10 μg/ml for SM. We calculated the concentrations at which 90% of the susceptible strains were inhibited (MIC_90_) and the 95% confidence intervals using the probit method.

### Inhibitory Activity against Mycolic Acid Biosynthesis

M. bovis BCG cell culture was apportioned to each assay tube at a volume of 0.98 ml, and then 0.01 ml of the test sample solution or DMSO (vehicle control) was added. Then, 0.01 ml of 2-^14^C acetic acid sodium salt was added to each tube at 1 mCi/tube (37 Bq/tube), followed by incubation at 37 °C for 60 min. The ^14^C-labeled cells were harvested by centrifugation at 2,000 × g for 10 min and hydrolyzed by 2 ml of 10% potassium hydroxide/methanol (20% potassium hydroxide:methanol = 1:1, vol/vol) at 37 °C for 1 h. After incubation, 1 ml of 6 M hydrochloric acid was added and mixed gently. Then, 5 ml of n-hexane was added, followed by extraction by shaking for 20 min. Separating upper-phase centrifugation (1,000 × g for 5 min) was then performed, and 4 ml of the upper hexane phase was removed and transferred to another tube and dried at 100 °C. For methyl esterization, 1 ml of benzene-methanol-concentrated sulfuric acid (10:20:1, vol/vol/vol) was added and incubated at 100 °C for 1 h for drying. Then, 0.2 ml of n-hexane was added and mixed to extract ^14^C-labeled fatty acid and mycolic acid. The extracted fatty acid and the mycolic acid subclasses were separated onto a thin-layer plate of Silicagel 60 F254 (thin-layer chromatography plate, Merck Japan; http://www.merck.co.jp/eng/index.html). 0.01 ml of extracted hexane phase was applied to the plate and allowed to develop to a diameter of 4 cm in the first solvent (heptan–diethylether–acetic acid [94:5:1, vol/vol/vol]) and 8 cm in the second solvent (petroleum ether–acetic acid [98:2, vol/vol]). Three thin-layer chromatography plates were fixed with an imaging plate (BAS-SR, Fujifilm; http://www.fujifilm.com) and analyzed by the following procedures: ^14^C-labeled fatty acid and mycolic acid were detected using a BAS-2500 imaging system (Fujifilm). The radioactivity of each mycolic acid subclass was calculated as photo-stimulated luminescence using Image Gauge software (Version 2.54).

Statistical analysis was conducted, using SAS software (R.8.1, SAS Institute; http://www.sas.com), on the values of percent of control that were calculated automatically using Image Gauge software (Version 2.54) based on the result of each photo-stimulated luminescence. The significance level of the test was set at 5%. IC_50_ values (concentration required to inhibit by 50%) and 95% confidence intervals were calculated by linear regression analysis with logarithmic transformed concentrations.

### Analysis of Metabolites Produced after Mixing OPC-67683 and M. bovis BCG Tokyo

15 μl of ^14^C OPC-67683 (0.5 mg/ml:1 μCi/μl) was added to 585 μl of 7H9/TN-ADC broth or bacterial culture and incubated for 48 h. After incubation, a 2-fold volume of acetonitrile was added and mixed well. The lysate was centrifuged for 5 min at 15,000 rpm, and the supernatant was analyzed using high-performance liquid chromatography (HPLC) with flow scintillation analyzer to determine the metabolite pattern. In a parallel experiment, 0.1 ml of the supernatant was added to the vial containing 5 ml of Scintillation Cocktail (Ultima Gold, Perkin Elmer; http://www.perkinelmer.com). The pellet was suspended in 600 μl of 2 M sodium hydroxide and incubated for 1 h at 60 °C, and 0.1 ml of the suspension was added to the vial containing the Scintillation Cocktail. These samples were measured using a Scintillation Counter (LS5000CE, Beckman; http:/www.beckmancoulter.com) to confirm the existence of covalently binding radioactive molecules.

### Determination of the Structure of Metabolite Produced after Mixing OPC-67683 and M. bovis BCG Tokyo

75 μl of OPC-67683 (0.5 mg/ml) was added to 2,925 μl of 7H9/TN-ADC broth or M. bovis BCG Tokyo bacterial culture and incubated for 72 h. After incubation, a 2-fold volume of acetonitrile was added and mixed well. The lysate was centrifuged for 5 min at 15,000 rpm, and the supernatant was then analyzed using LC-MS/MS to determine the structure of the detected metabolite produced by mixing OPC-67683 with M. bovis BCG Tokyo. The identified metabolite was synthesized at Otsuka Pharmaceutical, and the fragment pattern of the metabolite was then compared with that of another compound newly synthesized based on the predicted structure.

### Activity against Intracellular Mycobacteria

Human THP-1 monocytic cells were differentiated into macrophages by treatment with 100 ng/ml phorbol 12-myristae 13-acetate (PMA) in RPMI-1640 medium and were distributed at a portion of 1 × 10^6^/ml after a 2-d incubation. The differentiated macrophages were then inoculated with 6.88 log_10_ CFU of M. tuberculosis H37Rv for 4 h, washed twice with the medium to roughly remove the non-infecting bacteria, and then treated with 20 μg/ml SM for 20 h to kill the remaining viable extracellular bacteria. The starting CFU count in the cells was 6.42 log_10_ CFU. The cells were subsequently treated with the designated test compound for 4 h and were then washed twice with fresh medium to remove the added test compound. After an additional 68-h culture, the cells were lysed using 0.1% SDS, and the viable bacteria were counted in 7H11 agar plates to determine the potency against intracellular mycobacteria.

### Plasma Levels in an Experimental Mouse Model of TB

Mice were anesthetized by an intramuscular administration with a 0.05-ml solution containing ketamine and xylazine (Ketalar 50 [Sankyo; http://www.sankyo.co.jp/english]/Serakutaru 2% [Bayer; http://www.bayer.com])/sterile physiological saline solution = 8:3:9), infected by an intratracheal inoculation with a 0.05-ml cell suspension (1,010 CFU) of M. tuberculosis Kurono using feeding needle and micro-syringe, and housed for 28 d prior to the initiation of administration. The designated compound dissolved or suspended in 5% gum arabic was then administered orally. Blood samples (approximately 1 ml) at each time-point were collected into a heparinized syringe from the abdominal post cava under ether anesthesia. The blood samples were then centrifuged (3,000 rpm, at 5 °C) to extract the plasma. The plasma (0.1 ml) was mixed with acetonitrile (0.2 ml) for RFP and with ethanol (0.3 ml) for INH, EB, and PZA. For OPC-67683, the plasma obtained was filtered through a 0.22-μm filter, and then 0.1 ml of the filtered plasma was mixed with 0.5 ml of 0.5 M carbonate buffer (pH 10) and 5 ml of diethyl ether. After shaking for 10 min, the organic layer (4 ml) was dried using nitrogen gas at 40 °C and dissolved with 0.2 ml of methanol/water/formic acid (50/50/0.1). The samples were analyzed using HPLC and high-performance liquid chromatography–electrospray ionization–tandem mass spectrometry (LC-ESI-MS/MS).

### Therapeutic Efficacy

For evaluation of the therapeutic efficacy of OPC-67683, we designed three experiments that used various mouse models of TB, as described below. In each experiment, the designated compound dissolved or suspended in 5% gum arabic was administered orally once daily. At the end of the treatment period, the mice were euthanized (exsanguination through the abdominal inferior vena cava) under ether anesthesia, and the lung was aseptically excised. A lung homogenate for each mouse was prepared by pestling the lung evenly with a glass homogenizer after adding sterile distilled water to the excised lungs, and the homogenate was then diluted further with distilled water. A smear plate for each lung homogenate was then prepared by spreading 0.1 ml of each diluted solution on a 7H11 agar plate using a spreader. After spreading the homogenate solution, all plates were incubated at 37 °C and counted for formed colonies after 14 d.

#### Therapeutic efficacy in an experimental mouse model of chronic TB.

In order to examine the therapeutic efficacy of OPC-67683 and to determine the therapeutic dose range, an experimental mouse model of chronic TB was established by inoculating Institute of Cancer Research (ICR) mice with M. tuberculosis Kurono through the caudal vein and allowing the infection to develop for 28 d. OPC-67683, RFP, INH, EB, SM, or PZA was then administered once daily for 28 d to examine the change in viable bacterial count in the lung. ICR mice were inoculated intravenously with 8.6 × 10^4^ CFU of *M. tuberculosis* Kurono. After a 28-d period, the mice were assigned to groups (*n =* 5/group) using a stratified randomization method based on the body weight of each infected mouse. The test compounds were then administered orally once daily for 28 d (OPC-67683: 40 to 0.156 mg/kg, RFP: 20 to 1.25 mg/kg, INH: 20 to 1.25 mg/kg, EB: 160 to 20 mg/kg, SM: 160 to 20 mg/kg, PZA: 320 to 40 mg/kg, and PA-824: 40 to 1.25 mg/kg [2-fold dilutions]). CFU counts were performed as described above. All lungs were homogenized with 5 ml of sterile distilled water.

Statistical analysis was conducted using SAS software (R.8.1) on the number of viable bacteria in the lung of mice surviving until necropsy on the 57th day after inoculation, and on the number at the start of the treatment, which was on the 29th day after inoculation. The significance level of the test was set at 5%. A test for dose dependency was performed using linear regression analysis based on log-transformed values of the viable bacterial counts in the lung. When dose dependency was confirmed, the Williams' test (lower-tailed) was subsequently performed, and when dose dependency was not confirmed, the Dunnett's test (two-tailed) was subsequently performed against each of the control groups.

#### Therapeutic efficacy in an experimental TB model using immunocompromised mice.

To examine whether immunity relates to the mechanism of action in vivo, we performed experiments using BALB/c nude mice, which lack both conventional CD4^+^ and CD8^+^ T cells. The anti-tubercular activity of OPC-67683 in nude mice was compared with that in immunocompetent mice. BALB/c nude mice and BALB/c mice were inoculated intravenously with 2.04 × 10^4^ CFU of *M. tuberculosis* Kurono. 1 d after inoculation, the mice were assigned to groups (*n =* 5/group) using a stratified randomization method based on the body weight of each infected mouse. OPC-67683 was then administered orally once daily for 10 d (OPC-67683: 10 to 0.313 mg/kg [2-fold dilutions]). CFU counts were performed as described above. All lungs were homogenized with 5 ml of sterile distilled water.

#### Therapeutic efficacy in combination with conventionally used drugs.

A new regimen that included OPC-67683 was evaluated and compared with a global standard regimen to determine the best regimen for reducing the treatment duration in an experimental mouse model of chronic TB. ICR mice were inoculated intratracheally under anesthesia with 855 CFU of *M. tuberculosis* Kurono, and left for 28 d to allow the animals to develop chronic TB. Grouping (*n =* 6/group) was conducted by a stratified randomization method based on the body weight of each infected mouse. The test regimens were then administered orally for 2 mo in the combination of OPC-67683, RFP, and PZA, or RFP, INH, EB, and PZA as an intensive treatment, and for an additional 2 mo in the combination of OPC-67683 and RFP or 4 mo in the combination of RFP and INH as a maintenance treatment. The doses used in this experiment provided plasma levels in mice similar to those seen at the standard doses used in humans: for RFP, we used 5 mg/kg; for INH, 10 mg/kg; for EB, 100 mg/kg; and for PZA, 100 mg/kg. We set the dose for OPC-67683 at 2.5 mg/kg.

Necropsy was performed on days 29, 57, 85, 113, 141, 169, and 177 relative to the inoculation for the standard regimen and vehicle control groups and on days 29, 57, 85, 113, and 141 for the new-regimen groups. A lung homogenate for each mouse from a drug-treated group was prepared by pestling the lung evenly with a glass homogenizer after adding to the excised lungs 5 ml of sterilized distilled water on day 29 and 2 ml of sterilized distilled water on the day of necropsy. Lung homogenates for all vehicle control groups were prepared by pestling the lung evenly with a glass homogenizer after adding 5 ml of sterilized distilled water to the excised lungs. Smear plates of lung homogenate samples from the groups after 2–6 mo of treatment were prepared by spreading all of the lung homogenate on 7H11 agar plates.

Statistical analysis was conducted using SAS software (R.8.1) on the viable bacteria number in the lungs of mice surviving until necropsy after the inoculation. The significance level of the test was set at 5%. The viable bacterial count in the lungs of mice anatomized at days 57, 85, 113, and 141 were log-transformed for comparing the new regimen with the standard regimen using the two-tailed Dunnett's test. The mean values and 95% confidence intervals were calculated for evaluating the new regimen.

### In Vitro Metabolism of OPC-67683 in Human and Animal Liver Microsomes

The study was undertaken to investigate the metabolites produced by the metabolic reactions of OPC-67683 using human, rat, mouse, dog, rabbit, and monkey liver microsomes. Pooled human liver microsomes (20 mg/ml) from ten donors were prepared at the Biomedical Research Institute, Human and Animal Bridge Discussion Group (Chiba, Japan) [15]. Human liver samples were legally procured from the National Disease Research Interchange (http://www.ndriresource.org/) through the international partnership with the Human and Animal Bridge Discussion Group. The study was conducted in accordance with the Declaration of Helsinki.

The incubation mixtures contained 100 mM phosphate buffer (pH 7.4), 100 μM OPC-67683, 2.5 mM β-NADPH, 2.5 mM β-NADH, and 1 mg/ml microsomal protein in a final incubation volume of 0.5 ml. OPC-67683 was dissolved in DMSO, and the concentration of the organic solvent was 1% (v/v) in the reaction system. The reactions were performed in duplicate in a shaking water bath at 37 °C for 2 h. The incubation mixtures were extracted with acetonitrile and ethyl acetate, and the samples were analyzed by HPLC and LC-ESI-MS/MS.

### Effect of OPC-67683 on Cytochrome P450–Mediated Reactions in Human Liver Microsomes

7-ethoxyresorufin *O*-deethylase activity by CYP1A1/2, coumarin 7-hydroxylase activity by CYP2A6, 7-benzyloxyresorufin *O*-debenzylase activity by CYP2B6, tolbutamide methylhydroxylase activity by CYP2C8/9, S-mephenytoin 4′ -hydroxylase activity by CYP2C19, bufuralol 1′ -hydroxylase activity by CYP2D6, chlorzoxazone 6-hydroxylase activity by CYP2E1, and testosterone 6β-hydroxylase and nifedipine oxidized activities by CYP3A4 were determined as previously reported [16].

Standard incubation mixtures of 0.5 ml contained microsomal protein (0.1–0.5 mg), 0.1 M potassium phosphate buffer (pH 7.4), 0.1 mM EDTA, NADPH-generating system (2.5 mM β-NADP, 25 mM glucose-6-phosphate, 2 units of glucose-6-phosphate dehydrogenase, and 10 mM magnesium chloride), and substrates with or without OPC-67683. OPC-67683 was dissolved in DMSO and added to incubations at a volume of 5 μl. Substrates were dissolved in the following solvents: 7-ethoxyresorufin and 7-benzyloxyresorufin in DMSO; coumarin, bufuralol, and nifedipine in ethanol; tolbutamide, *S*-mephenytoin and testosterone in methanol; and chlorzoxazone in 1% (w/v) aqueous solution. The substrate solutions were added to incubations at a volume of 5 μl. The enzyme incubations were carried out in duplicate, and formations of metabolites were determined by HPLC.

Assay methods were validated in this study. The calibration curves were established for resorufin (0.2–200 nM, *r* = 0.9996), 7-hydroxycoumarin (0.05–5 μM, *r* = 0.9998), 4-hydroxytolbutamide (0.05–10 μM, *r* = 0.9998), 4-hydroxymephenytoin (0.025–5 μM, *r* = 0.9996), 1′ -hydroxybufuralol (0.025–5 μM, *r* = 0.9995), 6-hydroxychlorzoxazone (0.25–100 μM, *r* = 0.9994), 6β-hydroxytestosterone (0.03–30 μM, *r* = 0.9994), and oxidized nifedipine (0.1–25 μM, *r* = 0.9998).

7-ethoxyresorufin (0.5 μM), coumarin (2 μM), 7-benzyloxyresorufin (1.5 μM), tolbutamide (400 μM), *S*-mephenytoin (100 μM), bufuralol (20 μM), chlorzoxazone (100 μM), testosterone (100 μM), and nifedipine (50 μM) were selected as the concentrations of the substrates for the determination of residual activity in the presence of OPC-67683 (1–100 μM). The concentrations of the substrates were approximately the K_m_ values for the enzymes as previously reported [17]. Selective Cytochrome P450 inhibitors were used in this study to confirm the validity of the assays. 7,8-benzoflavone [18], furafylline [19], orphenadrine [20], quercetin [21], sulfaphenazole [22], tranylcypromine [23], quinidine [24], diethyldithiocarbamate [25], and ketoconazole [26], which are inhibitors of CYP1A1, 1A2, 2B6, 2C8, 2C9, 2C19, 2D6, 2E1, and 3A4, respectively, inhibited the respective enzyme activities. Diethyldithiocarbamate is also known to be a specific inhibitor of CYP2A6 [18], and the present study confirmed the potent inhibitory capability of this compound on CYP2A6-mediated metabolism.

### Other Information

The care and handling of the animals was in accordance with “Guidelines for Animal Care and Use in Otsuka Pharmaceutical Co., Ltd.” The aspects of experiments related to biosafety were performed according to standards set forth in “Biosafety manuals in Microbiological Research Institute and 3rd Institute of New Drug Discovery, Otsuka Pharmaceutical Co., Ltd.”

## Results

### BRM Test

The mutagenic potential of OPC-67683 was evaluated in the absence and presence of S9 mix using the BRM test in accordance with OECD Guideline 471. As shown in Table 1, OPC-67683 did not show mutagenicity.

**Table 1:**
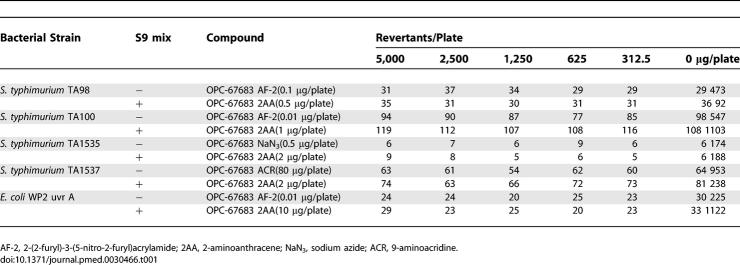
Bacterial Reverse Mutation Test for OPC-67683

### Susceptibility Testing

The MICs against standard strains are shown in Table 2. At concentrations ranging from 0.006 to 0.012 μg/ml, OPC-67683 inhibited the growth of both drug-susceptible and drug-resistant M. tuberculosis. The MICs of OPC-67683 were, respectively, four to 64, two to 32, 128 to 256, 64 to 512, eight to 16, and four to 16 times lower than those of RFP, INH, EB, SM, CGI-17341, and PA-824. These results indicate that OPC-67683 possesses the most potent anti-mycobacterial activity against both drug-susceptible and drug-resistant strains.

**Table 2:**
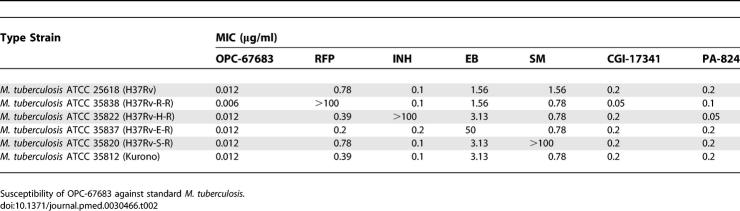
In Vitro Anti-Mycobacterial Activity of OPC-67683 Compared with RFP, INH, EB, SM, CGI-17341, and PA-824

The anti-tubercular activity was also evaluated on 67 clinically isolated strains. The MIC_90_ values (range) of OPC-67683, RFP, INH, EB, and SM were, respectively, 0.012 μg/ml (0.006–0.024 μg/ml), 0.288 μg/ml (0.05–0.78 μg/ml), 0.099 μg/ml (0.05–0.78 μg/ml), 3.636 μg/ml (0.78–6.25 μg/ml), and 2.938 μg/ml (0.39–6.25 μg/ml). Based on these results, the MIC_90_ values of OPC-67683 were about 24, eight, 303, and 244 times lower than those of RFP, INH, EB, and SM, respectively. The results of our evaluation indicated that OPC-67683 inhibited the growth of the clinically isolated drug-susceptible M. tuberculosis at the same range as on standard strains, and also showed activity against the clinically isolated strains resistant to the currently used anti-TB drugs RFP, INH, EB, or SM. These results indicate that OPC-67683 exhibits anti-mycobacterial activity on both drug-susceptible and drug-resistant strains and that it has no cross-resistance with any of the currently used anti-TB drugs. These data are shown in Table 3.

**Table 3:**
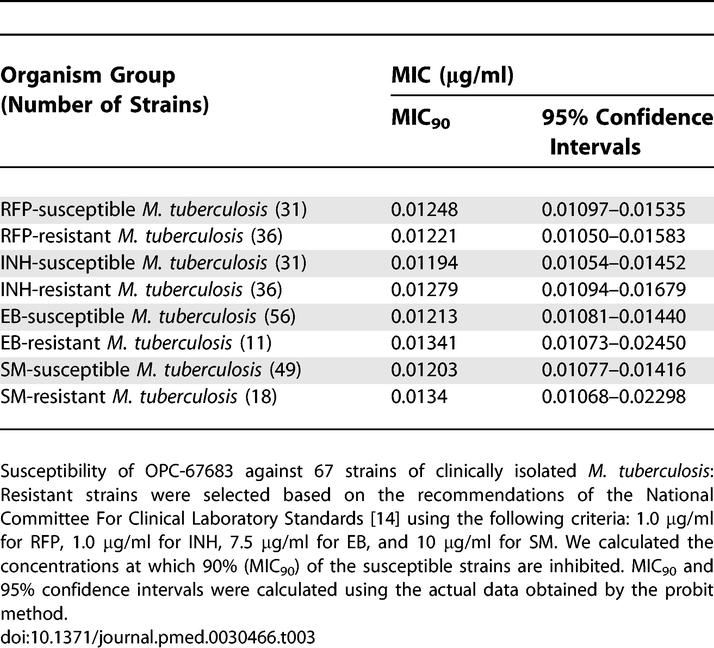
MIC_90_ of OPC-67683 against Drug-Susceptible and Drug-Resistant M. tuberculosis

In addition, the efficacy of OPC-67683 in combination with currently used anti-TB drugs RFP, INH, EB, and SM was examined in vitro using the checkerboard method. These results are shown in Table 4. The results showed OPC-676783 to have no antagonistic activity in combination with any of the drugs tested.

**Table 4:**
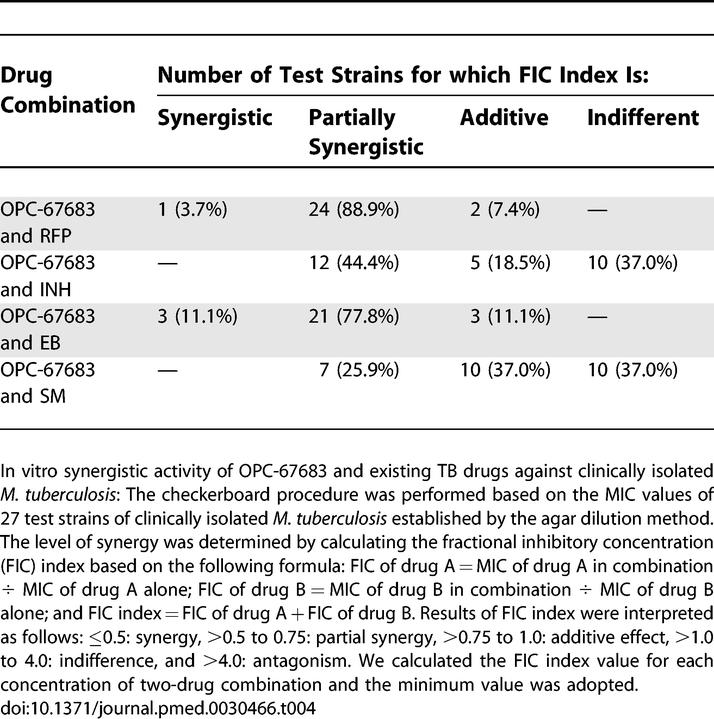
In Vitro Synergistic Activity of OPC-67683 and Existing TB Drugs against Clinically Isolated M. tuberculosis

### Inhibitory Activity against Mycolic Acid Biosynthesis

^14^C-labeled fatty acid and mycolic acid were detected using the BAS-2500 imaging system (unpublished data). The percent with respect to the control of each mycolic acid subclass was calculated automatically, and IC_50_ was calculated using SAS software. The results indicated that both OPC-67683 and INH inhibited mycolic acid synthesis, but the manner of action differed between the two compounds: OPC-67683 inhibited the synthesis of methoxy- and keto-mycolic acid, with IC_50_ values of 0.021 to 0.036 μg/ml, but not the synthesis of α-mycolic acid at concentrations up to 0.25 μg/ml, while INH inhibited all mycolic acid subclasses, with IC_50_ values of 0.630 to 1.851 μg/ml. The IC_50_ and 95% confidence interval values are shown in Table 5.

**Table 5:**
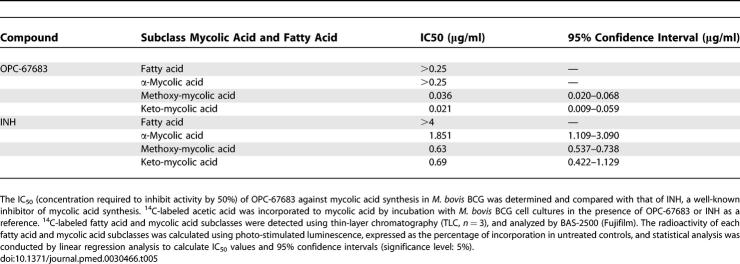
IC_50_ of OPC-67683 and INH against Mycolic Acid Synthesis

### Analysis of Metabolites Produced after Mixing OPC-67683 and M. bovis BCG

**Figure 2:**
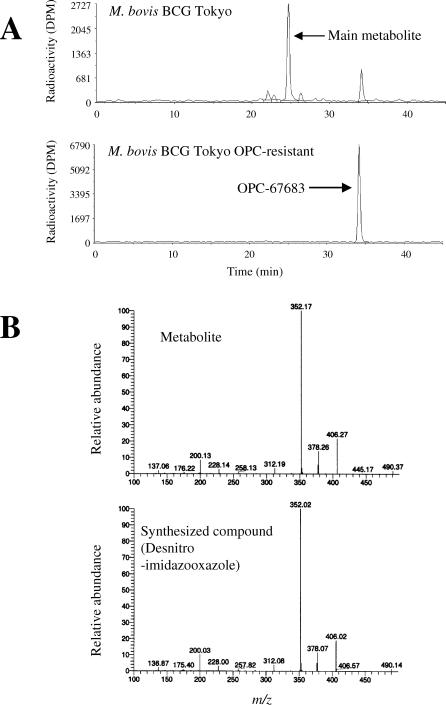
Analysis of Metabolites Produced after Mixing OPC-67683 and M. bovis BCG (A) 15 μl of ^14^C OPC-67683 (0.5mg/ml: 0.056 μCi/μl) was added to 585 μl of 7H9/TN-ADC broth or bacterial culture and incubated for 48 h. After incubation, a 2-fold volume of acetonitrile was added and mixed well. The lysate was centrifuged for 5 min at 15,000 rpm. The supernatant was analyzed using HPLC with flow scintillation analyzer to determine the metabolite pattern. (B) The identified metabolite (desnitro-imidazooxazole) was synthesized at Otsuka Pharmaceutical, and the fragment pattern of the metabolite by electrospray ionization mass spectroscopy was then compared with that of another compound newly synthesized based on the predicted structure.

After mixing OPC-67683 with M. bovis BCG Tokyo, we identified only one main metabolite, and this metabolite eluted faster than OPC-67683. No metabolites, however, were observed after mixing OPC-67683 with an experimentally obtained OPC-67683-resistant M. bovis BCG Tokyo strain. These results are shown in Figure 2A. The supernatant was analyzed using LC-MS/MS to determine the structure of the identified metabolite. We found the mass number of the identified metabolite to be 490 and predicted this structure to be a desnitro-imidazooxazole. We then synthesized a desnitro-imidazooxazole and performed a product ion scan with the identified metabolite and the newly synthesized compound. We observed product ions in 200, 352, 378, and 406 m/z in each experiment. Structural analysis of the main metabolite indicated that the structure was a desnitro-imidazooxazole possessing the same substituent as that of OPC-67683. The MS spectrum is displayed in Figure 2B.

In addition, when we treated the drug-susceptible strain with the radioactive OPC-67683, none of the radioactivity was recovered after the addition of acetonitrile. About 20% of the total radioactivity was distributed to the cell components, and this phenomenon was not observed with an OPC-67683-resistant strain. These data are shown in Table 6.

**Table 6:**
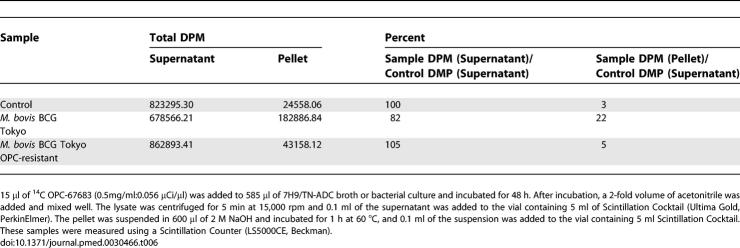
Analysis of OPC-67683-Susceptible and -Resistant M. bovis BCG Using Radio-Labelled OPC-67683

### Activity against Intracellular Mycobacteria in Human Macrophages

**Figure 3:**
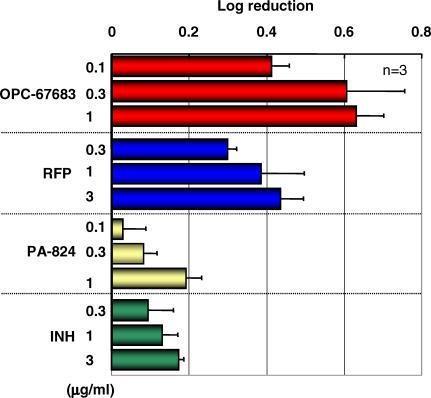
Effect of Pulsed Exposures to OPC-67683, RFP, INH, and PA-824 on the Intracellular Growth of M. tuberculosis H37Rv within THP-1 Cells Infected cells were incubated with the test compound for 4 h, washed, cultured until 68 h at 37 °C, plated on 7H11 agar, and counted for colonies after 16 d of growth at 37 °C. Values represent mean ± S.D (*n =* 3).

A study was conducted to confirm the post-antibiotic effect of OPC-67683 on intracellular M. tuberculosis in THP-1 cells, and the results were compared with RFP, INH, and PA-824. OPC-67683 was shown to be highly active against intracellular M. tuberculosis H37Rv after 4-h pulsed exposures in a dose-dependent manner. The data are shown in Figure 3. The intracellular activity of OPC-67683 at a concentration of 0.1 μg/ml was similar to that of RFP of 3 μg/ml, but was superior to INH and PA-824, which both showed poor activity during the 4-h pulsed exposure. These results indicated that even with limited contact with the bacteria within the cells, OPC-67683 might be able to effectively kill the intracellular mycobacteria.

### Plasma Levels in an Experimental Mouse Model of TB

**Figure 4:**
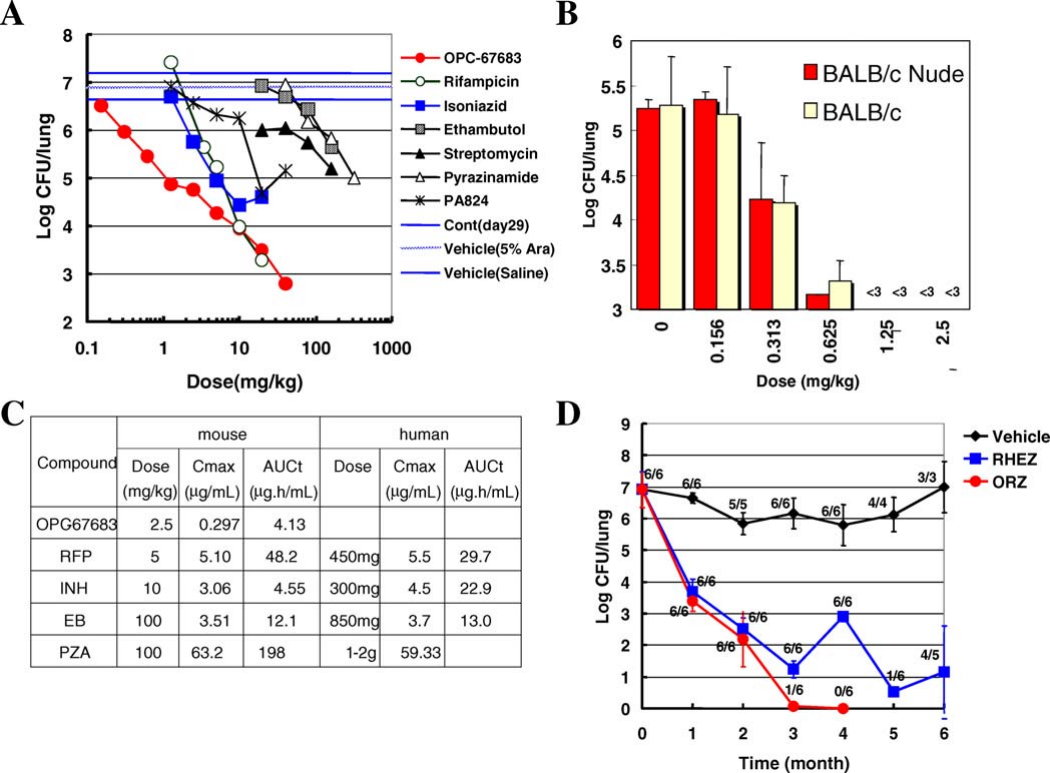
Effects of OPC-67683 in an Experimental Mouse Model of TB (A) ICR mice were inoculated intravenously with M. tuberculosis Kurono. After 28 d, test compounds were administered orally once daily for 28 d (OPC-67683: 40–0.156 mg/kg, RFP: 20–1.25 mg/kg, INH: 20–1.25 mg/kg, EB: 160–20 mg/kg, SM: 160–20 mg/kg, PZA: 320–40 mg/kg, and PA-824: 40–1.25 mg/kg; 2-fold dilution). Mean value (*n =* 5) of log_10_ CFU was plotted. (B) BALB/c standard and nude mice were inoculated intravenously with M. tuberculosis Kurono. From the following day, OPC-67683 was administered orally once daily for 10 d (OPC-67683: 10–0.313 mg/kg, 2-fold dilution). The bar was expressed as mean value and SD (*n =* 5) of log_10_ CFU. (C) The doses of conventional drugs used for evaluating regimen are summarized in this table. The doses set up for using the plasma C_max_ achieved in mice TB model is equivalent to that achieved in humans at the clinical dose. (D) ICR mice were inoculated intratracheally with M. tuberculosis Kurono. After 28 d, mice were treated for 2 mo with a combination of OPC-67683, RFP, and PZA (ORZ), or RFP, INH, EB, and PZA (RHEZ) (intensive treatment), and for an additional 2 mo with OPC-67683 and RFP or 4 mo with RFP and INH (maintenance treatment) (OPC-67683: 2.5 mg/kg, RFP: 5 mg/kg, INH: 10 mg/kg, EB: 100 mg/kg, and PZA: 100 mg/kg). Mean value and SD bar (*n =* 6) of log_10_ CFU was plotted. The fraction refers to the number of mice in which at least one colony was detected of the total number of surviving mice.

As shown in Table 7, OPC-67683 exhibited the lowest plasma concentration but longest half-life among the tested reference drugs. The C_max_ and AUC_t_ values for RFP, EB, and PZA in mouse plasma at the tested dose were similar to those in human at clinical doses. The C_max_ value for INH in mouse plasma was also similar to that in humans, but the AUC_t_ in the mouse was lower than that in humans. A comparison of these parameters between mouse and human plasma is summarized in Figure 4C [27–29].

**Table 7:**
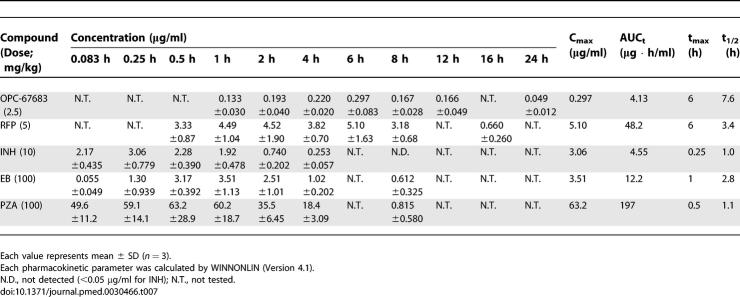
Plasma Concentration of OPC-67683, RFP, INH, EB, and PZA after Oral Administration in Mice Infected with M. tuberculosis Kurono

### Therapeutic Efficacy

#### Therapeutic efficacy in an experimental mouse model of chronic TB.

The viable bacterial count in the OPC-67683-treated groups decreased dose-dependently, and the therapeutic effects of the compound were observed and compared with those of the reference drugs. The results are shown in Figure 4A and Table S1. The dose groups that showed a significant decrease in pulmonary viable bacterial count when compared with the vehicle control group were 0.313, 0.625, 1.25, 2.5, 5, 10, 20, and 40 mg/kg for OPC-67683; 3.5, 5, 10, and 20 mg/kg for RFP; 2.5, 5, 10, and 20 mg/kg for INH; 160 mg/kg for EB, 20, 40, 80, and 160 mg/kg for SM; and 80, 160, and 320 mg/kg for PZA.

The doses of OPC-67683, RFP, INH, EB, SM, and PZA that could produce a CFU reduction of at least 95% in this experimental mouse model were 0.625, 3.5, 5, >160, 40, and 160 mg/kg, respectively.

#### Therapeutic efficacy in an experimental TB model using immunocompromised mice.

These results are shown in Figure 4B.

The pulmonary CFU counts of the OPC-67683-treated BALB/c nude mice and immunocompetent mice were reduced dose-dependently, and significant decreases were observed at doses of 0.313, 0.625, 1.25, and 2.5 mg/kg. The efficacy profiles of OPC-67683 were similarly excellent in both types of mice.

#### Therapeutic efficacy in combination with conventionally used drugs.

The eradication rate of a new regimen containing OPC-67683 was compared with that of the standard regimen. The OPC-67683-containing regimen exerted a rapid and consistent reduction during the first 3 mo (Figure 4D). At 3 mo after the start of treatment, only one colony was detected in one of the six animals; at 4 mo, no colonies were detected in any of the six animals. In contrast, at 6 mo for the standard regimen, colonies were detected in four out of five mice. These results suggest that a new regimen containing OPC-67683 could dramatically reduce the treatment duration by at least 2 mo.

### In Vitro Metabolism in Human and Animal Liver Microsomes

The current study was conducted to investigate the metabolites produced by in vitro metabolism of OPC-67683 using human and animal liver microsomes and to investigate the in vitro ability of OPC-67683 to affect the metabolism of substrates for CYP1A1/2, CYP2A6, CYP2B6, CYP2C8/9, CYP2C19, CYP2D6, CYP2E1, and CYP3A4. The results are shown in Table 8.

**Table 8:**
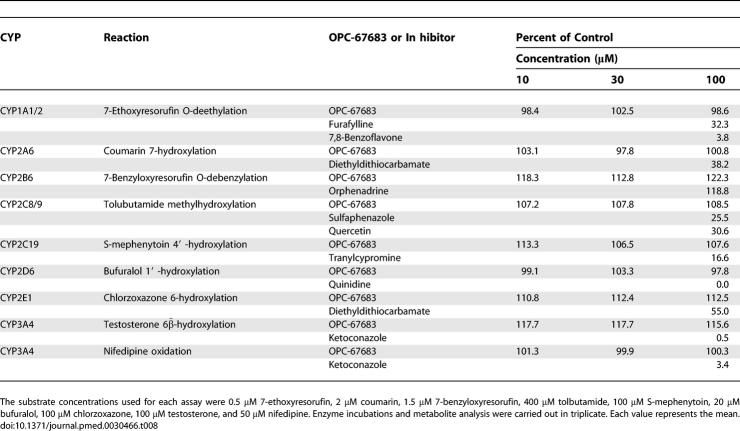
Effect of OPC-67683 on CYP1A1/2, CYP2A6, CYP2B6, CYP2C8/9, CYP2C19, CYP2D6, CYP2E1, and CYP3A4 Mediated Reactions in Human Liver

The HPLC and LC-ESI-MS/MS data demonstrated that the major metabolites were hardly detected in the incubation mixture OPC-67683 with human, rat, mouse, dog, rabbit, and monkey liver microsomes. OPC-67683 was stable in the in vitro metabolism of human and animal liver microsomes. These results suggest that OPC-67683 is not metabolized by the CYP enzymes.

OPC-67683 had neither stimulatory nor inhibitory effects on CYP1A1/2, CYP2A6, CYP2B6, CYP2C8/9, CYP2C19, CYP2D6, CYP2E1, and CYP3A4 activities at concentrations up to 100 μM, indicating that OPC-67683, at the expected therapeutic concentrations, would not be predicted to cause clinically significant interactions with other CYP-metabolized drugs.

## Discussion

With the several disadvantages to the current TB drug regimen, there are a number of expectations for a new anti-TB drug. An ideal new drug should be safe and able to shorten the treatment duration, be effective against MDR-TB, treat TB patients co-infected with HIV, and effectively address LTBI. We have performed our TB research program with these expectations in mind.

To shorten the duration of treatment, we focused our search on finding more powerful anti-TB agents, as history has shown that the introduction of more potent drugs can effectively reduce the required duration of treatment, as was the case with RFP and PZA. For improved efficacy against MDR-TB, we screened for compounds with a new structure and mechanism of action. Furthermore, to target LTBI, we focused on compounds with activity against intracellular M. tuberculosis.

Mycobacteria are well known to be wax-rich bacteria, and a main component of the wax is mycolic acid, which is detected only in mycobacteria and not in gram-positive or gram-negative bacteria or in mammalian cells. Genome research of tubercle bacilli has verified this lipid richness, showing there to be almost 250 distinct enzymes involved in the lipid metabolism of tubercle bacilli [30]. In view of the important role of mycolic acid in mycobacteria, we searched for a compound that could inhibit mycolic acid synthesis and demonstrate potent anti-TB activity in vitro. We found OPC-67683 to have both inhibitory activity on mycolic acid biosynthesis and potent in vitro activity against M. tuberculosis, as indicated by its low MIC range across many strains, including MDR-TB. The IC_50_ values of OPC-67683 for mycolic acid subclasses were lower than those of INH, and these results correlated well with the in vitro anti-tubercular activity of OPC-67683 and INH. The anti-tubercular activity of nitro-imidazooxazole derivatives correlated well with their inhibitory activity against mycolic acid biosynthesis [11]. We therefore concluded that the inhibitory activity of OPC-67683 against mycolic acid synthesis was a mechanism of action attributable to killing mycobacteria at least as potently as INH.

As M. tuberculosis can grow not only facultatively but also as intracellular organisms that survive and multiply in macrophages of the infected host, we consider it important that a compound is also able to kill intracellular TB and that such activity should correlate with a shortened treatment duration and could be an important factor in the treatment of LTBI. We therefore examined the killing activity against intracellular TB in macrophage-derived THP-1 cells. Among the tested compounds, OPC-67683 demonstrated the most potent killing activity. The killing activity of OPC-67683 at 0.1 μg/ml was similar to that of RFP at 3 μg/ml and was superior to that of INH and PA-824. The intracellular potency of antibiotics is commonly evaluated in vitro using continuous exposure rather than in animal models due to their often-rapid elimination, depending on the plasma half-life. OPC-67683 was able to demonstrate potent in vitro killing ability even at short exposure times. These results indicate that OPC-67683 would likely exert strong antibiotic activity against intracellular TB in patients even at short exposure times, which could be an advantage in intermittent treatment.

PA-824 has been reported to be a prodrug metabolized to its active form by mycobacterium [31]. Recently, Manjunatha et al reported that Rv3547 acts as the catalytic enzyme for PA-824, but the role of Rv3547 within mycobacterium is not yet clear [32]. Similarly, OPC-67683 also requires metabolic activation by M. tuberculosis in order for the anti-TB activity to be exerted. Experimentally isolated OPC-67683-resistant mycobacterium did not metabolize the compound. We confirmed a mutation in the Rv3547 gene among the resistant organisms, indicating Rv3547 to be a key enzyme involved in activating OPC-67683, as it was for PA-824 (unpublished data). According to Manjunatha et al, the metabolites of PA-824 have not yet been identified. With OPC-67683, however, the main metabolite produced in the presence of M. tuberculosis was identified as a non-active desnitro-imidazooxazole. This result suggests that Rv3547 possesses a reduction potency of the nitro residue and that an intermediate between OPC-67683 and the desnitro-imidazooxazole could be the active form. After mixing radioactive OPC-67683 with viable mycobacterium, nearly 20% of the radioactive substances were not recovered. In contrast, after treating OPC-67683-resistant mycobacterium, nearly 100% of radioactivity was recovered. The action mechanism of metronidazole derivatives against H. pylori has been reported to be due to the production of a radical intermediate [33]. This information suggests the possibility that a radical intermediate that appears as the intermediate for the metabolism of a nitro residue covalently binds to the target molecule. If this hypothesis is correct, it could well explain the strong post-antibiotic effect seen with OPC-67683 against intracellular mycobacterium, a property considered necessary to kill latent TB.

The therapeutic efficacy of OPC-67683 was evaluated in vivo in an experimental chronic TB mouse model. In this model, OPC-67683 exhibited the most potent anti-tubercular activity in comparison with the reference compounds. The viable bacterial counts in the lung were markedly reduced dose-dependently by OPC-67683 at 0.313 mg/kg and higher. A 95% reduction in bacterial load was achieved at 0.625 mg/kg. Furthermore, the efficacy of OPC-67683 in a TB model established using immunodeficient mice was similar to that seen using standard mice.

Treatment of TB requires combination therapy not only to shorten the treatment duration but also to prevent the development of resistance. The effects of OPC-67683 in combination with currently used TB drugs were therefore evaluated both in vitro and in vivo. OPC-67683 did not exert antagonistic effects in any of the tested combinations, and produced partial synergistic or synergistic effects when combined with RFP or EB in vitro. A combination regimen containing OPC-67683, RFP, and PZA produced a steady rapid reduction in bacterial load over the first 3 mo. These results suggest that a new regimen containing OPC-67683 could possibly be effective in shortening the clinical treatment duration.

Multiple-drug therapy is a common clinical practice, particularly in patients with concomitant diseases or conditions. However, whenever two or more drugs are administered concurrently, the possibility of drug interactions exists. Many drug interactions are clinically caused by inhibition of drug-metabolizing enzymes, such as CYPs, leading to decreased metabolic clearance and increased exposure to the inhibited drug [34–36]. Rifamycin derivatives such as RFP usually induce CYP3A4 enzymes, remarkably reducing the bioavailability of the drug itself as well as other CYP-intermediated drugs, including protease inhibitors, which are indispensable in the treatment of HIV/AIDS [37]. It is therefore important that a new TB drug does not induce nor is affected by metabolic enzymes. With this in mind, we studied the interactions between OPC-67683 and metabolic enzymes. Our results showed that OPC-67683 was hardly metabolized when exposed to human and animal liver microsomes and did not have inductive, stimulatory, or inhibitory effects on CYP enzyme activities at concentrations up to 100 μM, indicating that OPC-67683, at the expected therapeutic concentrations, would not be expected to cause clinically significant interactions with other CYP-metabolized drugs, such as rifamycin derivatives. These results, together with data supporting non-compromised anti-TB activity in immunodeficient animals, suggest that OPC-67683 could be useful in treating TB patients who are also co-infected with HIV/AIDS.

We conclude that OPC-67683 possesses qualities that could help address the unmet needs in TB chemotherapy, i.e., the need for shortened treatment duration, effectiveness against MDR-TB, ability to be used safely in HIV/AIDS patients, and the treatment of LTBI. An early Phase II clinical study to confirm the efficacy in patients is now ongoing.

Furthermore, the Global Alliance for TB Drug Development is aiming to establish an entirely new regimen containing the best combination of new drugs [38]. Development and integration of these drugs into the regimen individually would normally be done in series, taking at least six years for each drug. We therefore attach importance to including an evaluation of the effects of OPC-67683 in combination with not only conventional drugs but also new drugs as early as possible in order to contribute data necessary for establishing the best regimen needed to address the unmet needs in TB treatment.

## Supporting Information

Table S1 Viable Count in Lung of Each Group of OPC-67683, RFP, INH, EB, SM, PZA, and PA-824 after 4 wk of Treatments on the Experimental Chronic TB Model in Mice (43 KB DOC)

## Abbreviations

BRM: bacterial reverse mutation
CFU: colony-forming unit
DMSO: dimethylsulfoxide
EB: ethambutol
HPLC: high-performance liquid chromatography
ICR: Institute of Cancer Research
INH: isoniazid
LTBI: latent tubercle bacilli infection
MDR-TB: multidrug-resistant tuberculosis
MIC: minimum inhibitory concentration
PZA: pyrazinamide
RFP: rifampicin
SM: streptomycin
TB: tuberculosis
