# Supplementary material for: OPC-67683, a Nitro-Dihydro-Imidazooxazole Derivative with Promising Action against Tuberculosis In Vitro and In Mice
Source: PLoS Med. 2006 Nov 28;3(11):e466. doi: 10.1371/journal.pmed.0030466 (PMC1664607; doi:10.1371/journal.pmed.0030466)
Supplement: Table S1 — (43 KB DOC) [file pmed.0030466.st001.doc]

Supplemental Table 1. Viable Count in Lung of Each Group of OPC-67683, RFP, INH, EB, SM, PZA, and PA-824 after 4 Weeks Treatments on the Experimental Chronic Tuberculosis Model in Mice

|  | Dose(mg/kg) | N= | Log of Viable Counts/Lung^#1^ |
| --- | --- | --- | --- |
| Cont(day29)^#2^ | 0 | 5 | 6.634 ± 0.303 |
| Vehicle(5% Ara)^#3^ | 0 | 5 | 6.889 ± 0.280 |
| Vehicle(Saline)^#3^ | 0 | 5 | 7.187 ± 0.407 |
| OPC-67683 ^#4^ | 0.156 | 5 | 6.506 ± 0.294^a:NS, b:NS^ |
|  | 0.313 | 5 | 5.959 ± 0.436^a:**, b:**^ |
|  | 0.625 | 5 | 5.457 ± 0.245^a:**, b:**^ |
|  | 1.25 | 5 | 4.867 ± 0.400^a:**, b:**^ |
|  | 2.5 | 5 | 4.753 ± 0.297^a:**, b:**^ |
|  | 5 | 5 | 4.272 ± 0.463^a:**, b:**^ |
|  | 10 | 5 | 3.943 ± 0.465^a:**, b:**^ |
|  | 20 | 5 | 3.495 ± 0.522^a:**, b:**^ |
|  | 40 | 5 | 2.791 ± 0.671^a:**, b:**^ |
| Rifampicin ^#5^ | 1.25 | 5 | 7.409 ± 0.201^c:NS, d:NS^ |
|  | 3.5 | 5 | 5.634 ± 0.371^c:*, d:*^ |
|  | 5 | 5 | 5.231 ± 0.1.150^c:**, d:**^ |
|  | 10 | 5 | 3.972 ± 0.596^c:**, d:**^ |
|  | 20 | 5 | 3.279 ± 0.330^c:**, d:**^ |
| Isoniazid ^#5^ | 1.25 | 5 | 6.702 ± 0.237^c:NS, d:NS^ |
|  | 2.5 | 5 | 5.751 ± 0.358^c:**, d:*^ |
|  | 5 | 5 | 4.944 ± 0.760^c:**, d:**^ |
|  | 10 | 5 | 4.434 ± 0.319^c:**, d:**^ |
|  | 20 | 5 | 4.595 ± 0.452^c:**, d:**^ |
| Ethambutol  ^#4^ | 20 | 5 | 6.923 ± 0.721^a:NS, b:NS^ |
|  | 40 | 5 | 6.696 ± 0.382^a:NS, b:NS^ |
|  | 80 | 5 | 6.429 ± 0.475^a:NS, b:NS^ |
|  | 160 | 5 | 5.642 ± 0.120^a:**, b:**^ |
| Streptomycin^#4^ | 20 | 5 | 6.000 ± 0.553^a:**, b:*^ |
|  | 40 | 5 | 6.040 ± 0.361^a:**, b:*^ |
|  | 80 | 5 | 5.739 ± 0.309^a:**, b:**^ |
|  | 160 | 5 | 5.184 ± 0.461^a:**, b:**^ |
| Pyrazinamide^#4^ | 40 | 5 | 6.942 ± 0.205^a:NS, b:NS^ |
|  | 80 | 5 | 6.168 ± 0.774^a:*, b:NS^ |
|  | 160 | 5 | 5.823 ± 0.628^a:**, b:*^ |
|  | 320 | 5 | 5.009 ± 0.539^a:**, b:**^ |
| PA-824 ^#5^ | 1.25 | 5 | 6.905 ± 0.382^c:NS, d:NS^ |
|  | 2.5 | 5 | 6.567 ± 0.108^c:NS, d:NS^ |
|  | 5 | 5 | 6.329 ± 0.303^c:NS, d:NS^ |
|  | 10 | 5 | 6.244 ± 0.728^c:NS, d:NS^ |
|  | 20 | 5 | 4.688 ± 0.944^c:**, d:**^ |
|  | 40 | 5 | 5.145 ± 0.444^c:**, d:**^ |

#1: Each value represented mean ± S.D..

#2: Cont(day29) was the control mice at the day 29 that represented the initial viable before treatments.

#3: Vehicle(5% Ara) and Vehicle(Saline) were the control mice at the day 57 treated with vehicles( 5% Gum Arabic, and Saline).

#4: Dose dependency (Regression Analysis: Linearity ; P<0.01, Lack of Fit ; Not Significant

#5: Dose dependency (Regression Analysis : Linearity ; P<0.01, Lack of Fit ; P<0.05

a : Williams test ( Lower-tailed ) vs Vehicle(5% Ara) : * ; P<0.05, ** ; P<0.01, NS ; Not Significant

b : Williams test ( Lower-tailed ) vs Cont(day29) : * ; P<0.05, ** ; P<0.01, NS ; Not Significant

c : Dunnett test( Two-tailed ) vs Vehicle(5% Ara) : * ; P<0.05, ** ; P<0.01, NS ; Not Significant

d : Dunnett test( Two-tailed ) vs Cont(day29) : * ; P<0.05, ** ; P<0.01, NS ; Not Significant
